# Mapping heatwave health risk at the community level for public health action

**DOI:** 10.1186/1476-072X-11-38

**Published:** 2012-09-13

**Authors:** Camille Buscail, Erika Upegui, Jean-François Viel

**Affiliations:** 1 Department of Epidemiology and Public Health, University Hospital, Rennes, France; 2 CNRS n° 6249 “Chrono-Environment”, Faculty of Medicine, Besançon, France; 3 INSERM n° 1085, “Epidemiological Research on Environment, Reproduction and Development”, Faculty of Medicine, Rennes, France

**Keywords:** Heatwave health risk, Urban heat island, Vulnerable populations, Spatial risk assessment, Remote sensing, Land surface temperature, Land cover, Public health

## Abstract

**Background:**

Climate change poses unprecedented challenges, ranging from global and local policy challenges to personal and social action. Heat-related deaths are largely preventable, but interventions for the most vulnerable populations need improvement. Therefore, the prior identification of high risk areas at the community level is required to better inform planning and prevention. We aimed to demonstrate a simple and flexible conceptual framework relying upon satellite thermal data and other digital data with the goal of easily reproducing this framework in a variety of urban configurations.

**Results:**

The study area encompasses Rennes, a medium-sized French city. A Landsat ETM + image (60 m resolution) acquired during a localized heatwave (June 2001) was used to estimate land surface temperature (LST) and derive a hazard index. A land-use regression model was performed to predict the LST. Vulnerability was assessed through census data describing four dimensions (socio-economic status, extreme age, population density and building obsolescence). Then, hazard and vulnerability indices were combined to deliver a heatwave health risk index. The LST patterns were quite heterogeneous, reflecting the land cover mosaic inside the city boundary, with hotspots of elevated temperature mainly observed in the city center. A spatial error regression model was highly predictive of the spatial variation in the LST (*R*^*2*^ = 0.87) and was parsimonious. Three land cover descriptors (NDVI, vegetation and water fractions) were negatively linked with the LST. A sensitivity analysis (based on an image acquired on July 2000) yielded similar results. Southern areas exhibited the most vulnerability, although some pockets of higher vulnerability were observed northeast and west of the city. The heatwave health risk map showed evidence of infra-city spatial clustering, with the highest risks observed in a north–south central band. Another sensitivity analysis gave a very high correlation between 2000 and 2001 risk indices (*r* = 0.98, *p* < 10^-12^).

**Conclusions:**

Building on previous work, we developed a reproducible method that can provide guidance for local planners in developing more efficient climate impact adaptations. We recommend, however, using the health risk index together with hazard and vulnerability indices to implement tailored programs because exposure to heat and vulnerability do not require the same prevention strategies.

## Background

Research has shown a significant warming of global temperatures over the last 150 years [1]. If emissions of greenhouse gases continue unabated, temperatures are projected to rise between 1.1°C and 6.4°C above 1990 levels by the end of the century [2]. This warming would be accompanied by many types of extreme events, including heatwaves, which are forecast to increase in intensity, frequency and duration in the coming years [3,4].

Cities and urban areas tend to be hotter than rural areas, especially at night, creating urban heat islands (UHIs) whose effects are exacerbated during a heatwave [5-7]. The UHI effects are due to a range of factors, including increased absorption and reflection of the sun on concrete compared to green or brown spaces (with more of the sun’s energy being stored in urban surfaces during the day and released into the atmosphere at night); reduced cooling from breezes due to airflow obstruction from buildings; and anthropogenic heat release from industry, businesses and transport [8]. Moreover, UHI effects worsen air quality by increasing the formation of secondary pollutants such as ozone [9].

Climate change is increasingly acknowledged as a serious threat to population health. A number of observational studies conducted across Europe, the USA, Canada and Australia, have shown an association between high temperatures and all-cause, cardiovascular and respiratory mortality [10-16]. People living in inner city areas that are subsequently exposed to the effects of UHI and air pollution are at increased heat-related health risk [17-19]. Human vulnerability to heatwaves results from a set of risk factors [20-22], although death also occurs among the fit and healthy during a severe and/or prolonged heatwave.

Among socio-demographic factors, extreme age (children younger than five and the elderly) is associated with an increased health risk [23-26]. Other risk factors include social isolation, low income or immigration [27-29]. Education level also seems to modify the heat-mortality relationship. Individuals with at most a high school education have higher death rates during heatwaves [27,30]. People living alone, regardless of their age, have been found to be at increased heat risk in the USA [31-33] and, to a lesser extent, Europe [15,34]. Married people are less likely to die from heat compared with those who are widowed, divorced or never married [14,15,20]. Chronic or severe illnesses represent other vulnerability factors to heat. Individuals unable to care for themselves, with limited mobility or suffering from respiratory, cardiovascular or neurological diseases are at high risk during a heatwave [11,24,34,35].

Physical environment factors, such as building and housing features, also contribute to differences in heat-related health risk. People who live in south-facing top-floor flats, in old buildings or in high-rise buildings are more vulnerable than those who do not [19,32]. High population density correlates with areas of higher temperatures through incoming solar radiation [36]. However, one must keep in mind that most of the studies performed on heatwave consequences in cities focused on mega-cities such as Chicago, New York or Paris, which are too unique to have results that can be easily generalized.

Climate change poses unprecedented challenges, ranging from global and local policy challenges to personal and social action. Heat-related deaths are largely preventable but interventions for the most vulnerable populations need improvement. Surveillance and alert for heat-related conditions are usually only conducted at the national or regional level. This resolution lacks sufficient spatial detail to better inform planning and prevention, making intra-urban heat risk assessment of paramount importance. In working on the frontlines with communities, local authorities play crucial roles as key communicators and influencers of the public in their areas in addition to developing localized prevention programs. During a heatwave, local authorities and social care services can ensure that health and social care workers have identified those most at risk from a heatwave in their community. Then, they can arrange, where appropriate, for a daily visit/phone call by a formal or informal caregiver (e.g., family, neighbor, friend, volunteer or community sector worker). In the longer term, local authorities can implement policies to change the built environment or to lower anthropogenic emissions.

Reducing the impact of heatwaves requires, therefore, the prior identification of geographical areas most in need of intervention. In this paper, our aim was to assess heat-related health risk at the small-area level to help cities target their resources in a cost-effective way. To make this method easily transferable to any urban area exposed to heatwaves, we studied a medium-sized town and relied upon satellite thermal data with other digital data, thereby avoiding any field-collection efforts.

## Methods

### Study area

The study area encompasses the city of Rennes (Brittany, France). Spread over 50.36 km^2^ and containing 207,922 inhabitants (2007), this city can be considered representative of medium-sized cities on the French scale.

French census geographic entities have a structured hierarchy. We chose the lowest level, block groups (or “IRIS” in French), equivalent to census block groups in the USA or the British lower super output areas, as the working level. The city of Rennes is divided into 92 IRIS, with a surface area ranging from 0.06 to 4.71 km^2^ (mean, 0.55 km^2^), and a population from 3 to 4,287 inhabitants (mean, 2,260 inhabitants).

### Conceptual framework

Following Tomlinson et al. [37], we used the risk framework developed by Crichton [38] that appeared particularly appropriate to climate change studies [39-41]. Risk is described as a function of hazard, exposure and vulnerability. If any one of those elements is missing, there is no risk. In the case of heatwaves, the hazard is the increase in temperature. Exposure refers to the inventory of elements at the location at which hazard events may occur. A typical indicator of exposure to heatwaves is population census data from the areas affected by the hazard. Vulnerability is the absence of material or social resources to cope with or mitigate the effects of extreme heat. This factor depends to a large degree on individual risk factors (e.g., age over 65) but also on building characteristics (e.g., high-rise buildings). The final risk map is generated from the combination of the hazard index on the one hand, and the exposed and vulnerable index on the other. Because of French privacy laws and confidentiality requirements, all items of interest were merged at the IRIS level. To combine heterogeneous data and to facilitate interpretation of the data for local authorities, all original and combined variables were scaled by linear transformation, yielding hazard, vulnerability and risk indices lying between zero and one.

### Hazard

Atmospheric UHIs are usually detected by ground-based air temperature measurements taken from standard meteorological stations. However, these stations are often located in sparsely inhabited areas and the existing networks are inadequate for estimating the temperature gradient, making these data unrepresentative of the local heat experienced in residential settings.

Remote sensing satellites are therefore increasingly used to assess the thermal exposure of a population during a heatwave [42-44]. Satellite thermal data can depict the spatial gradient of radiometric surface (and not ambient) temperature [45]. Rather than employing a medium scale sensor (such as a 1.1 km resolution Advanced Very High Resolution Parameter [AVHRR] image from the U.S. National Oceanic and Atmospheric Administration satellite [NOAA]), the land surface temperature (LST) was estimated from Landsat Enhanced Thematic Mapper (ETM+) images acquired during the day, when heat island intensities are greatest. The ETM + thermal band 6 (10.4 to 12.5 μm) has a spatial resolution of 60 m at the nadir, which is considered to be suitable for capturing complex intra-urban surface temperature differences, thus allowing an effective and detailed analysis of the urban climate [42]. We used an image acquired on June 22, 2001, at 10:43 AM Universal Time Coordinated (UTC). This period was chosen because a localized heatwave (with a maximum mean daily temperature of 31.6 °C) hit the city of Rennes at that time (i.e., two years before the August 2003 major heatwave that struck Western Europe). Landsat revisit times average 16 days, so the collection of another image during this event was not possible. In a sensitivity analysis, we also considered a Landsat ETM + image acquired on July 21, 2000 (at 10:44 AM UTC), when there was not a heatwave.

The thermal band image data calibration was performed in a two-step process. The digital number (DN) values of band 6 were first converted into spectral radiance L [W / (m^2^ sr μm)] via a radiative transfer equation using gain and bias values recently reassessed by Chander et al. [46]:

$$\text{L}=\left(\text{G}\;*\;\text{DN}\right)+\text{B}$$

Where G (gain) = 0.037205, and B (bias) = 3.16.

Then, the radiance values of all pixels were converted to at-sensor brightness temperature (BT) in Kelvin (K) under the assumption of uniform emissivity using the following formula:

$$BT=\frac{K_{2}}{\ln\left(K_{1}/L+1\right)}$$

where K_2_ is the calibration constant (1282.71 K), and K_1_ is the calibration constant [666.09 W / (m^2^ sr μm)].

Finally, the LST was obtained by converting K degrees into C degrees (K = C + 273.5), and pixel values were spatially averaged at the IRIS level.

The satellite thermal remote sensing technique in the study of urban climates requires high-resolution images with a sufficient spatial coverage as well as an experienced analyst to interpret them. To overcome these potential limitations, land-use regression modeling represents an alternative way of estimating the LST. These models are attempts to better estimate exposure levels for a given population by using geographic predictor variables associated with the exposure under scrutiny. Originally developed as a means to assess exposures from traffic-related air pollution, they have become widely adopted as a method of describing the spatial variation of environmental threats, including heatwaves, because temperature levels are strongly associated with types of land cover [46-48]. We developed a land-use regression model of local surface temperatures at the IRIS level using cadastral data at a 0.5 m resolution downloaded from the city of Rennes website [49] (to calculate the proportions of surface covered by vegetation, water, buildings and streets) and the normalized difference vegetation index (NDVI) derived from Landsat bands 3 (red: 0.63 to 0.69 μm) and 4 (near-infrared: 0.75 to 0.90 μm) as independent variables. The percent vegetation for each IRIS was calculated as the proportion of land areas classified as parks or woods. Vegetation and water proportions were log-transformed to approximate normality. To explore LST drivers, we performed a spatial regression model based on the algorithm used by Anselin and Smirnov [50]. Contiguity-based spatial weights (first order queen contiguity to determine neighboring IRIS as those that have any point in common, including both common boundaries and common corners) were used. All explanatory variables with a *P* value < 0.20 in the univariate analysis were included in the final multivariate model.

Land-use regression models were performed with GeoDa software (version 1; GeoDa Center, Tempe, AZ).

### Exposure

Where there are no people, there is no exposure, and consequently, there is no risk. Moreover, a scarcely populated IRIS would yield unstable and unreliable vulnerability estimates. For both reasons, we decided a priori to ascribe a null value to the exposure, vulnerability and risk indices of the four IRIS inhabited by fewer than 200 people.

### Vulnerability

We considered four vulnerability dimensions (socio-economic status, extreme age, population density and building obsolescence) that have been demonstrated to modify the relationship between heat and health outcomes in the literature. Census data provided information about the spatial distribution of the required vulnerability characteristics at the IRIS level.

**Figure 1:**
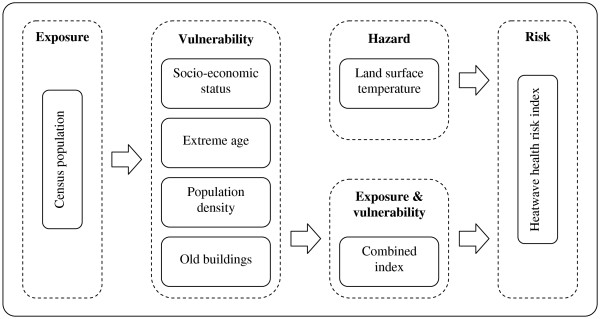
**Flowchart of the spatial risk assessment framework (adapted and developed from**[37]**).**

For socio-economic status, we merged three components: deprivation, social isolation and low education (each weighted at 33.3%) (Figure 1). We relied upon the Townsend score (one of the most widely used deprivation indices) [51] to assess the contextual economic level of each IRIS. The higher the Townsend index score, the more deprived and disadvantaged an IRIS is thought to be. Four variables (unemployed residents as a percentage of all economically active residents; households that do not own a car as a percentage of all households; households that do not own a home as a percentage of all households; and household overcrowding, i.e., more than one person per room) were extracted from the population census and combined to form an overall score for each IRIS. The proportion of single households was used as a proxy for social isolation. We assessed low educational level through the proportion of the population with no high school diploma.

Extreme age was characterized by the proportion of children younger than 5 and the proportion of people over 65 (both weighted at 50%). The density of inhabitants per IRIS was derived from their respective populations and surface areas. Finally, the proportion of inhabitants living in buildings built before 1975 defined building obsolescence because this threshold was a risk factor in a study conducted after the 2003 French heatwave [52].

### Risk

As shown in Figure 1, the four vulnerability dimensions were combined into a single exposed and vulnerable index (each weighted at 25%) and then were combined with the hazard index (each weighted at 50%) to deliver a heatwave health risk, expressed as an index varying between zero and one.

### Mapping

Spatial variations of hazard, vulnerability and risk indices were visualized using choropleth maps. The index ranges (from 0 to 1) were split into five equal intervals reflecting increasing (very low, low, moderate, high and very high) hazards, vulnerabilities or health risks. Maps were created and analyzed with Quantum GIS (version 1.7.4; Open Source Geospatial Foundation Project) and R software (version 2.12.2; R development Core team, Vienna, Austria).

## Results

**Figure 2:**
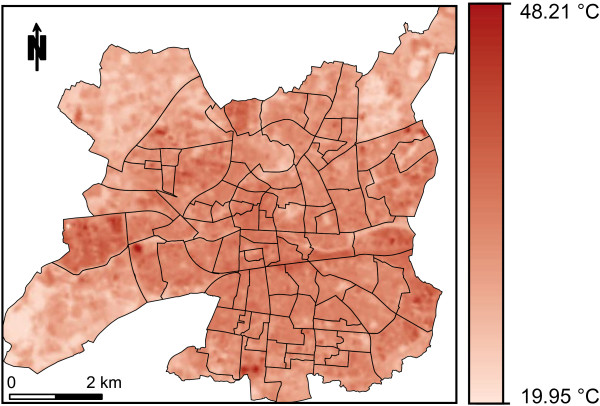
Land surface temperature map under heatwave conditions at the pixel level (June 22, 2001, at 10:43 AM UTC from a Landsat ETM + image, city of Rennes, France).

**Figure 3:**
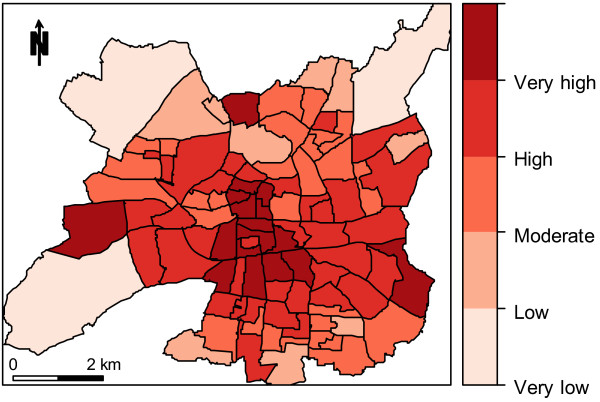
Hazard index map under heatwave conditions at the IRIS level (June 22, 2001, at 10:43 AM UTC from a Landsat ETM + image, city of Rennes, France).

Under heatwave conditions, the LST varied from 19.95 to 48.21°C at the pixel level (Figure 2), and from 26.45 to 34.75°C at the IRIS level. The hazard index (or scaled LST) distribution at the IRIS level is presented in Figure 3. Surface temperature patterns are quite heterogeneous, reflecting the land cover mosaic inside the city boundary. Instead of a single heat island, hotspots of elevated temperature are mainly observed in the city center. The lowest temperatures are observed in three IRIS mainly covered by parks and located at the city’s edge.

Table 1 presents the descriptive statistics of the explained and predictive variables used to build the land-use regression model. The regression diagnostics revealed high spatial autocorrelation and guided towards a spatial error model. Predictors for surface temperature are summarized in Table 2. With three explanatory variables, the spatial error regression model explains a very high proportion of the variation in the LST (*R*^*2*^ = 0.87). The Akaike information criterion (AIC) decrease relative to non-spatial model (from 208.80 to 180.91) reflects the improvement of fit for the spatial error specification. The Moran’s I statistic for residuals of 0.04 indicates that all spatial autocorrelation is eliminated. NDVI, vegetation and water correlate negatively with surface temperature, with NDVI having the strongest effect (*p* < 10^-12^). The sensitivity analysis (based on the Landsat ETM + image acquired one year earlier) yields similar results (Table 2), with an *R*^*2*^ equal to 0.80, a decrease in AIC (from 177.15 to 166.11), and a Moran’s I statistic for residuals of 0.01.

**Table 1:** Descriptive summary of the factors included in the land use regression model (92 IRIS, city of Rennes, France)

**Variable**	**Minimum**	**Mean**	**Median**	**Maximum**	**Standard deviation**
LST^a^ (°C)	26.45	31.59	31.74	34.75	1.64
NDVI^b^	−0.30	−0.04	−0.03	0.18	0.10
Vegetation (%)	0.00	0.05	0.00	0.68	0.11
Water (%)	0.00	0.02	0.00	0.21	0.03
Buildings (%)	0.01	0.22	0.21	0.58	0.10
Streets (%)	0.03	0.14	0.15	0.27	0.05

^a^ Land surface temperature.

^b^ Normalized difference vegetation index.

**Table 2:** Land surface temperature predictors (92 IRIS, city of Rennes, France)

**Parameters**	**Heatwave Landsat image (June 22, 2001)**			**Non-heatwave Landsat image (July 21, 2000)**		
	**β**	**SE**	**p**	**β**	**SE**	**p**
Intercept	31.18	0.22	<10^-12^	29.42	0.14	<10^-12^
NDVI^a^	−14.02	1.06	<10^-12^	−10.33	0.90	<10^-12^
Water^b^	−27.03	5.31	<10^-6^	−23.47	5.07	<10^-5^
Vegetation^b^	−6.42	1.95	<10^-3^	−4.90	1.88	<10^-2^
Lambda^c^	0.70	0.09	<10^-12^	0.53	0.12	<10^-5^

^a^ Normalized difference vegetation index.

^b^ Log-transformed.

^c^ Spatial autoregressive error term.

**Figure 4:**
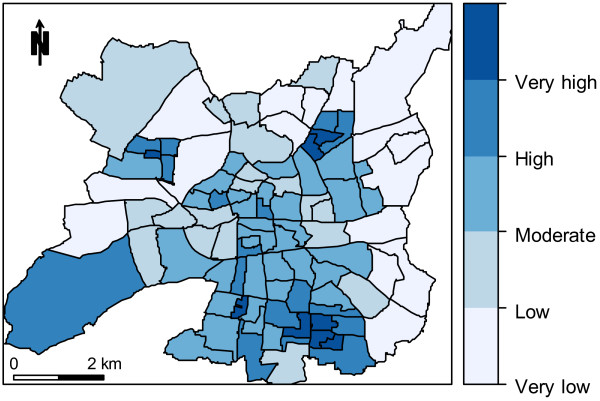
Vulnerability index map at the IRIS level (1999 census data, city of Rennes, France).

The southern areas show the most vulnerability, although some pockets of higher vulnerability are observed northeast and west of the city, broadly matching the disadvantaged neighborhoods (Figure 4). There is a general trend towards lower vulnerability in the north and east outskirts.

**Figure 5:**
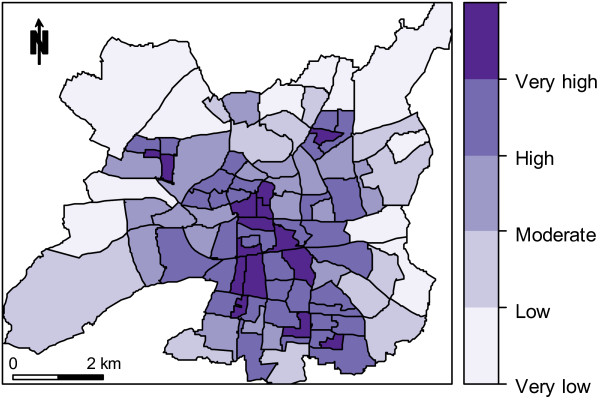
Heatwave health risk map at the IRIS level (city of Rennes, France).

Figure 5 displays the heatwave health risk and shows evidence of infra-city spatial clustering. The highest risks are observed in a north–south central band (including downtown areas). We obtained a similar pattern when using July 2000 temperature estimates in the risk index construction (map not shown), with a very high Spearman correlation coefficient between the 2000 and 2001 risk indices (*r*_*s*_ = 0.98, p < 10^-12^).

## Discussion

This study builds on previous work to demonstrate a simple and flexible conceptual framework that may serve as a template for future heatwave risk maps. A single cloud-free Landsat ETM + image (acquired whatever the warm season) or three land cover descriptors provide estimates of surface temperature. Vulnerability is assessed through the combination of various census data. Then, an intra-urban health risk index is constructed to address the needs for locally relevant projections of the potential effects of a heatwave on public health.

The strengths of this study include the Landsat ETM + imagery, the sensitivity analyses and the land-use regression model. By employing high resolution sensors (60 m resolution, much more suitable for small-scale urban temperature mapping than the 1.1 km resolution of NOAA AVHRR data), surface temperature estimates could be provided for any location and intra-urban gradients could be explored. Sensitivity analyses show that the remotely sensed image does not needed to be acquired during a heatwave (provided it is taken during summer time to avoid seasonal fluctuation [53]), confirming the robustness of the methodology based on relative LST for the prediction of heatwave health risk in a European urban area. The land-use regression model is based on sound statistical methods that account for spatial autocorrelation and on refined physiographic features of the landscape (precise surface geometry information enabling, for example, the discrimination of residential from commercial land and the identification of urban green space). The resulting model is characterized by parsimony and a high predictive capacity.

Some limitations must be considered in interpreting our results. Our approach does not explore the ways in which urban populations adapt physiologically and/or technologically to heat. Instead, it focuses on factors beyond individuals that can contribute to differing levels of risk and are amenable to preventive solutions in the long term (e.g., modifications of the built environment such as tree and shrub planting, heat-resistance roofing and paving material or the preservation of open space) [54].

The discrepancy of the data collection dates (1999 census data, 2000–2001 Landsat imagery and 2009 surface geometry data) inevitably creates some temporal ambiguity in index estimates. This circumstance should not, however, be of major concern, as the city of Rennes is characterized by a stable population (206,229 inhabitants in 1999; 207,922 inhabitants in 2007); therefore, land cover components and census data can also be considered relatively stable over time.

Disadvantages of Landsat imagery are its limited thermal calibration and its daytime collection (resulting from the near polar Sun-synchronous Landsat orbit) [55]. Nighttime images are considered to represent the UHI situation more accurately because during the night sensed thermal infrared radiance is restricted to only emissions from the ground (due to the cessation of direct solar radiation) [56]. Conversely, during the daytime, LST under shadow is lower than LST in direct sunlight, giving rise to steeper temperature gradients. This phenomenon most likely explains the wide range of LST values observed at the pixel level.

When calculating the LST from Landsat images, we assumed homogeneous atmospheric interference because this study was interested in relative LST over a spatial extent of a few kilometers (the error potentially produced is, therefore, uniform across the image) [42,53]. We also assumed uniform emissivity while emissivity differences between land cover types may induce inaccuracies when LST variations are examined [42].

Finally, two vulnerability variables could not be considered, although they are described in the literature. First, pre-existing health conditions may lead to susceptibility to heat-related illnesses and death. Unlike previous studies performed in the UK [37] and the USA [54], we have not included health perception or disease burden data in the vulnerability index because these data are unfortunately unavailable at an infra-city scale due to strict French privacy laws. Second, air conditioning is a strong protective factor against heat-related death [57], but it is not recorded in French census databases. However, air conditioning prevalence is much lower in France than in the USA, entailing very low contrasts across IRIS groups and making this variable less meaningful in our study.

Although the relation between remote-sensed LST and ambient temperature is not fully understood and remains mainly empirical [55,58], using satellite images in the thermal infrared band for defining UHIs is now common practice [42]. Few researchers have, however, mapped the potential impact of heatwaves following an integrated approach. Reid et al. provided heat vulnerability maps for census tracts scattered across the USA, using demographic characteristics, household air conditioning variables, vegetation cover from satellite images (30 m resolution) and the prevalence of diabetes [54]. Johnson et al. predicted the occurrence of death from extreme heat at the census tract level (Philadelphia, PA, USA) by integrating socio-demographic risk factors with LST estimates (120 m spatial resolution) [59]. Kestens et al. modeled the LST with meteorological data, the distance to major water bodies, NDVI, land cover, geographical coordinates, and the week of the year [48]. Following Tomlinson et al., who proposed a spatial risk assessment methodology to highlight potential heat health risk areas (Birmingham, UK) [37], we have attempted to identify the areas experiencing the hottest temperatures (but using higher resolution satellite imagery) and the most vulnerable populations in the community for quantifying risk at the small-area level, while envisaging alternative approaches when remotely sensed images are either acquired during a regular summer or are missing.

If no remote sensing image or skilled image analyst is available, land-use regression represents a valuable alternative to remote-sensed LST, although the spatial variation of urban LST is a complex issue and is subject to many factors [53,60,61]. We were able to describe a highly predictive and parsimonious spatial error regression model. The relationship between NDVI and LST is well established, but our study goes one step further by considering various characteristics of urban surfaces, whose combined effects have been less explored [62]. Using three basic land cover characteristics (NDVI, water, vegetation), we were able to construct a highly predictive land-use regression model, accurately quantifying LST gradients across an urban area. The NDVI and vegetation fraction are both vegetation indicators. However, the NDVI can be influenced by many factors external to the plant leaf (e.g., viewing angle, soil background) and does not provide areal estimates of the amount of vegetation [47,60]. Our results confirm NDVI as the dominant contributor (in agreement with Kestens et al. [48]). They also highlight the independent contributions of vegetation and water fractions in explaining the spatial variation of temperatures in urban areas, in line with Weng et al. [61]. Moreover, the interpretation of these results is straightforward: irrigated vegetation and water bodies cool the surroundings due to increased evaporation [62]. Notably, impervious surface fractions (as assessed by the proportions of surface covered by buildings or streets) were not predictive for LST, although they played an important role in modulating urban variability of LST in other studies [48,53,61,62]. Extrapolation of our land-use regression models to other study areas should, therefore, be cautious.

The sensitivity analysis provides clues that the LST prediction model may not be necessarily calibrated for extreme heat events, as regression estimates and goodness-of-fits are quite stable between 2000 and 2001, attesting to the robustness of our method. A single day snapshot appears sufficient, meaning that relative temperatures (i.e., spatial gradients) rather than absolute temperatures are required. In the absence of remote sensed data, NDVI data can be downloaded from the NASA website, but at a 1.8 km resolution [63]. Moreover, in case there is no precise local information about the land cover distribution of the studied area, the Corine Land Cover 2006 database can provide reliable and comparable information on land cover across Europe at a 250 m resolution [64].

Our vulnerability index includes data on both community properties (e.g., deprivation) and population composition (e.g., the proportion of elderly residents). All dimensions and indices were weighted equally. Weightings can, however, be easily modified according to new knowledge on heat-related health issues or to specific local authority requirements. The majority of the “very high” risk IRIS are grouped together in the city center, where the highest temperatures are experienced, as well as the highest proportion of people over 65, the highest proportion of isolated people, the highest proportion of old buildings and the highest population density. Other hotspots correspond to high-rise social housing and poorer communities. Obviously, these very high-risk areas are prime candidates for heat warning and heat reduction resource programs.

## Conclusions

Heatwaves can occur in any community. We used knowledge from previous epidemiological research to develop a simple method relying on pre-existing digital data, with the vision of easily reproducing the framework in a variety of urban configurations. The resulting risk map can provide guidance for local planners to develop more efficient climate impact adaptations by facilitating better resource allocation. We recommend, however, using the risk index together with hazard and vulnerability indices (and even with high resolution underlying databases) to identify which dimension contributes the most to health risk for a given area. Tailored programs could therefore be implemented because exposure to heat and vulnerability do not require the same prevention strategies.

## Abbreviations

AVHRR: Advanced Very High Resolution Radiometer; DN: Digital Number; ETM: Enhanced Thematic Mapper; LST: Land Surface Temperature; NDVI: Normalized Difference Vegetation Index; NOAA: National Oceanic and Atmospheric Administration; UHI: Urban Heat Island.

## Competing interests

The authors declare that they have no competing interest.

## Authors’ contributions

CB participated in the design of the study, performed the statistical analysis, and helped to draft the manuscript. EU analyzed the remote sensing images and estimated land surface temperatures. JFV conceived of the study, participated in its design and drafted the manuscript. All authors have read and approved the final manuscript.
